# Improved Spatial Registration and Target Tracking Method for Sensors on Multiple Missiles

**DOI:** 10.3390/s18061723

**Published:** 2018-05-27

**Authors:** Xiaodong Lu, Yuting Xie, Jun Zhou

**Affiliations:** Institute of Precision Guidance and Control, Northwestern Polytechnical University, Xi’an 710072, China; luxiaodong@nwpu.edu.cn (X.L.); zhoujun@nwpu.edu.cn (J.Z.)

**Keywords:** spatial registration, 3-D sensors, target tracking, Kalman Filter on Earth-Centered Earth-Fixed (ECEF-KF) coordinate algorithm, Pseudo Linear Kalman Filter (PLKF), Unscented Kalman Filter (UKF), error compensation

## Abstract

Inspired by the problem that the current spatial registration methods are unsuitable for three-dimensional (3-D) sensor on high-dynamic platform, this paper focuses on the estimation for the registration errors of cooperative missiles and motion states of maneuvering target. There are two types of errors being discussed: sensor measurement biases and attitude biases. Firstly, an improved Kalman Filter on Earth-Centered Earth-Fixed (ECEF-KF) coordinate algorithm is proposed to estimate the deviations mentioned above, from which the outcomes are furtherly compensated to the error terms. Secondly, the Pseudo Linear Kalman Filter (PLKF) and the nonlinear scheme the Unscented Kalman Filter (UKF) with modified inputs are employed for target tracking. The convergence of filtering results are monitored by a position-judgement logic, and a low-pass first order filter is selectively introduced before compensation to inhibit the jitter of estimations. In the simulation, the ECEF-KF enhancement is proven to improve the accuracy and robustness of the space alignment, while the conditional-compensation-based PLKF method is demonstrated to be the optimal performance in target tracking.

## 1. Introduction

The spatial registration is vital for current and near-future cooperative combat missions through the vehicle network to estimate and compensate the sensor errors by measuring the common target [1,2,3].

Depending on the dimension of the error model, the algorithm is usually divided into a two-dimensional (2-D) system and a three-dimensional (3-D) system [4]. Many typical 2-D algorithms have been proposed in literatures. e.g., Real Time Quality Control (RTQC) [5], Least Square (LS) [6], Generalized Least Square (GLS) [7], Exact Maximum Likelihood (EML) [8]. Compared to 3-D algorithms, the former wherein has relatively lower computational complexity at the expense of accuracy. Meanwhile, a number of online registration algorithms for 3-D sensors have been put forward. Unfortunately, there are few approaches taking both measurement errors and orientation errors into consideration. The traditional Earth-Centered Earth-Fixed coordinate (ECEF) method [9] capable of solving the spatial registration problem concentrates on the measurement biases, yet ignores the attitude errors of sensors themselves when the measurement states are transformed from separate reference frame to public system. The neglect of measurement deviations in the Kalman-filter-based method [10] proposed for attitude biases makes it deficient way as well. Although the algorithm [11] combines the two types of errors for estimation, it only fits for the situation where the attitude angles are small and the sensors are close to each other.

Besides, there is another branch of research that integrates the registration with target tracking. The paper [12] presents an improved method based on the state value and space deviation of federated filtering of unscented Kalman filter and standard Kalman filter, conducting registration and tracking simultaneously. However, this method is thought to accounts for more computation [13]. For this reason, the processes of those two are usually performed separately in current investigation. Moreover, most methods referred above are only suitable for sensors on stationary base which would be deemed points in the relative motion between them and targets. Failure of them to adapts for the sensors having their own attitude and position changes (e.g., radar seeker on high-speed moving and turning missiles) is considered as the deficiency.

Target tracking, based on sensor measurement information, is a technique for estimation of the state (position, speed) of a moving target at the current or future time [14,15]. Filtering is the cornerstone of tracking algorithm with the existence of systematic and measured noise. The typical filters have been greatly adopted for more than half a century: Kalman Filter (KF) is first exploited by [16] to solve the problem of tracking for linear system. Extended Kalman Filter (EKF) [17] transforms nonlinear model to linear one and then takes KF to estimate the states, which easily diverges under strongly nonlinearity. On this basis, [18] puts forwards Unscented Kalman Filter (UKF) which is apt for both linear and nonlinear tracking, but compromised by the heavy computation. To make up the disadvantage of the methods above, the Pseudo Linear Kalman Filter (PLKF) [19] which takes the models in [20] preserves the nonlinearity in the system. With the development of computers and data links during the last ten years, tracking performance is increased through the design and implementation of systems using data collected by a network of spatially distributed sensors. Consequently, the spatial registration has gradually become the pre-requisite for information process and data fusion for multiple sensors.

In the case of two missiles cooperative target tracking, this paper employs an improved Kalman Filter on Earth-Centered Earth-Fixed coordinate (ECEF-KF) algorithm which transforms the measurements to public target from body systems into the ECEF system so as to isolate the motion of sensors. Taking advantage of the difference of transformed values, the systemic biases and the attitude errors are estimated simultaneously. Subsequently, the results of registration algorithm adopted are fed back to compensate the inputs of linear PLKF and nonlinear UKF which are used for target tracking. It is found in this paper that the convergence of ECEF-KF and the effectiveness of compensation depend on the quality of the 3-D measurement data that can be adversely affected by the movement of missiles and target. More specifically, the 3-D information may loses one degree of freedom on any dimension and turns to be 2-D with the approach of vehicles, ruining the registration results. For sake of the possible divergence, a Low-pass First Order Filter (LFOF) with position-judgement condition is introduced to detect and inhibit divergent trend, furtherly improves the accuracy of the target tracking. The paper researches theoretically in the information processing methods, which conduces furtherly to the application of sensor in the engineering problems.

The main contributions are listed below:

1. A new spatial registration algorithm is first proposed for sensors on high-speed moving vehicles, realizing the simultaneous estimation for system and attitude biases which are compensated to the biased measurements of the tracking schemes. The accuracy and robustness of the estimation of target state are effectively improved.
2. Inspired by the ideal of integral controller, a Low-pass filter is used when the position relationship between missiles and target meets the special condition to inhibit the jitter of estimations. This skill improve the adaptability of tracking system without time-delay caused by the common integral controllers.

This paper is organized as follows. The definition of each coordinate system and their transform relationships are given in Section 2. Section 3 develops the model of original and improved ECEF-KF algorithm. The compensation-based PLKF and UKF methods with strategies of divergence detection and inhibition is introduced in Section 4. Section 5 presents the performance of improved ECEF-KF under two cases of motion of missiles and compares PLKF methods and UKF methods. The concluding remarks are given in Section 6.

## 2. Coordinate Defination and Transformation

Before the model development, the reference frames will be introduced in this section.

### 2.1. Definition of Coordinate System

**Figure 1:**
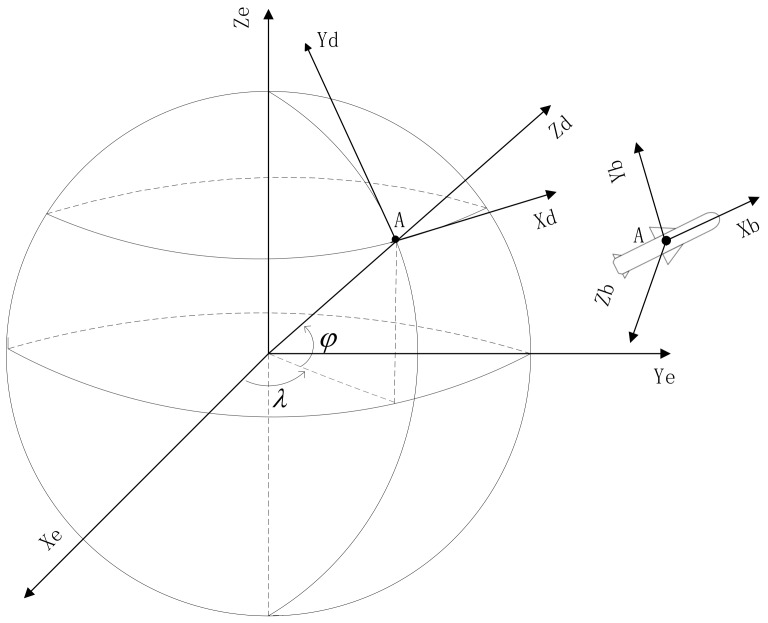
The definition of coordinate systems.

In the spatial alignment of multiple missiles, each member has its own reference frame or coordinate system. Among which, the measurements of sensors on the platform are obtained in the body coordinate system, the attitude angles and the position are usually described in the Local East, North, Up system and ECEF coordinate system respectively. The definition of these coordinate systems referred are listed as follows (see in Figure 1).

(1) ECEF Coordinate $OX_{e}Y_{e}Z_{e}$: As defined in WGS-84 coordinate [21], the origin is located in the earth’s Center of Mass (CM). The $Z_{e}$ axis points to the direction of Conventional Terrestrial Pole (CTP). The $X_{e}$ axis points to the intersection point of the zero-meridian plane and the CTP equator defined in Bureau International deI’Heure (BIH) 1984 [22]. The $Y_{e}$ axis and the $Z_{e}$ axis are perpendicular to the $X_{e}$ axis, forming the right-hand coordinate system.

(2) Local East, North and Up (LENU) Coordinate $OX_{d}Y_{d}Z_{d}$: The origin is the projection of the platform’s CM to the surface of the earth, the $X_{d}$, $Y_{d}$ and $Z_{d}$ axis points to the east, north and up respectively [23].

(3) Body Coordinate $OX_{b}Y_{b}Z_{b}$: The origin is located in the CM of platform. The $X_{b}$ axis coincides with the longitudinal axis of platform, pointing to the head as positive. The $Y_{b}$ axis coincides with the vertical axis of platform, pointing to the up as positive. And the $Z_{b}$ is determined by the right-hand rule.

### 2.2. Transformation between Reference Frames

(1) ECEF and LENU Coordinate: Let the column vectors $r_{e}={\left[\begin{array}{ccc} x_{e} & y_{e} & z_{e} \end{array}\right]}^{T}$ and $r_{d}={\left[\begin{array}{ccc} x_{d} & y_{d} & z_{d} \end{array}\right]}^{T}$ (the superscript T denotes matrix transposition) represent the ECEF coordinate and LENU coordinate respectively. The transformation from the later to the former is given by

(1) $$r_{e}=C_{d}^{e}r_{d}$$

where $C_{d}^{e}$ is the 3×3 orthogonal matrix given by [24]

(2) $$\left[\begin{array}{ccc} -\sin\lambda & -\sin\varphi \cos\lambda & \cos\varphi cos\lambda \\ \cos\lambda & -\sin\varphi \sin\lambda & \cos\varphi \sin\lambda \\ 0 & \cos\varphi & \sin\varphi \end{array}\right]$$

λ and φ denote the longitude and latitude of platform. Besides, if the longitude λ, latitude φ and height *h* are known, its ECEF coordinate can be calculated by [25]

(3) $$\begin{array}{c} x_{e}=\left(C+h\right)\cos\left(\varphi \right)\cos\left(\lambda \right)\\ y_{e}=\left(C+h\right)\cos\left(\varphi \right)\sin\left(\lambda \right)\\ z_{e}=\left(C\left(1-e^{2}\right)+h\right)\sin\left(\varphi \right)\\ C=R_{e}/\sqrt{1-e^{2}\sin^{2}\left(\varphi \right)}\; \end{array}$$

where $R_{e}$ is the equatorial radius, *e* is the eccentricity of the earth.

(2) LENU and Body Coordinate: Let the column vectors $r_{b}={\left[\begin{array}{ccc} x_{b} & y_{b} & z_{b} \end{array}\right]}^{T}$ represents the body coordinates. Likewise, the transformation from the body coordinate to the LENU coordinate is represented by a matrix $C_{b}^{d}$ in [26]

(4) $$\left[\begin{array}{ccc} \cos\theta \cos\psi & \sin\theta & -\cos\theta \sin\psi \\ -\sin\theta \cos\psi \cos\gamma +sin\psi sin\gamma & \cos\theta \cos\gamma & \sin\theta \sin\psi \cos\gamma +\cos\psi sin\gamma \\ \sin\theta \sin\gamma \cos\psi +\cos\gamma sin\psi & -\cos\theta \sin\gamma & -\sin\theta \sin\psi sin\gamma +\cos\gamma \cos\psi \end{array}\right]$$

where ψ, θ and γ are the yaw, pitch and roll angle of the platform respectively.

## 3. Registration Algorithm

The algorithm is based on the following assumptions:

- The attitude biases and the measured deviations are considered as constant and the attitude biases are assumed as small values.
- The coupling between biases is ignored.
- The position errors of sensors are not considered. It means the positions are known exactly with other possible assistant device (e.g., GPS).

### 3.1. Attitude and Sensor Measurement Errors

The ECEF-KF algorithm is introduced to estimate attitude and sensor measurement biases which are modeled as additive constant biases to the reported values. That is

(5) $$\left\{\begin{array}{c} {\tilde{\psi }}_{k}=\psi_{k}\;+\Delta \psi_{k}\\ {\tilde{\theta }}_{k}=\theta_{k}\;+\Delta \theta_{k}\\ {\tilde{\gamma }}_{k}=\gamma_{k}\;+\Delta \gamma_{k} \end{array}\right.$$

where ${\tilde{\psi }}_{k}$, ${\tilde{\theta }}_{k}$, ${\tilde{\gamma }}_{k}$ are the reported values of the *k* th sensor’s yaw, pitch and roll angles. $\psi_{k}$, $\theta_{k}$, $\gamma_{k}$ are the true values and $\Delta \psi_{k}$, $\Delta \theta_{k}$, $\Delta \gamma_{k}$ are the bias errors of these angles. Likewise, the measurement erros are modeled as

(6) $$\left\{\begin{array}{c} {\tilde{r}}_{k}\;=r_{k}\;+\Delta r_{k}\\ {\tilde{\alpha }}_{k}=\alpha_{k}+\Delta \alpha_{k}\\ {\tilde{\beta }}_{k}=\beta_{k}+\Delta \beta_{k} \end{array}\right.$$

where $r_{k}$, $\alpha_{k}$, $\beta_{k}$ represent the true values of the *k*th sensor’s slope distance (range), the elevation and azimuth angle of line of sight respectively. It is notable that the measurement errors include constant biases and random noises, that is

(7) $$\left\{\begin{array}{c} \Delta r_{k}\;=\Delta r_{km}\;+\Delta r_{kn}\\ \Delta \alpha_{k}=\Delta \alpha_{km}+\Delta \alpha_{kn}\\ \Delta \beta_{k}=\Delta \beta_{km}+\Delta \beta_{kn} \end{array}\right.$$

where $\Delta r_{km}$, $\Delta \alpha_{km}$, $\Delta \beta_{km}$ are the constant biases, and $\Delta r_{kn}$, $\Delta \alpha_{kn}$, $\Delta \beta_{kn}$ denote the random noises.

### 3.2. Traditional ECEF-KF Algorithm

**Figure 2:**
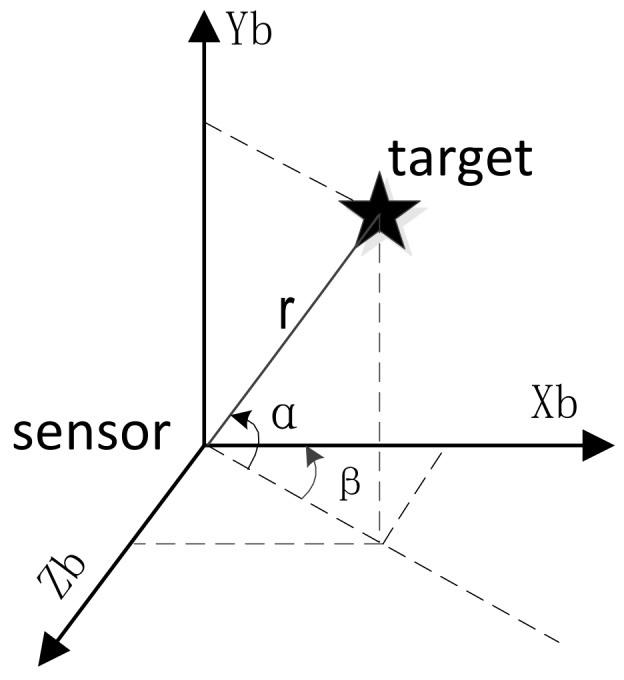
Measurement of target in body coordinate system.

Given the true measurements of range *r*, elevation α and azimuth β of radar sensor which are defined under body system, the non-linear relationship between target state $\left(x_{b},y_{b},z_{b}\right)$ and measurement is (see in Figure 2)

(8) $$\begin{array}{c} \alpha =\arcsin\frac{y_{b}}{\sqrt{x_{b}^{2}+y_{b}^{2}+z_{b}^{2}}}\\ \beta =-\arctan\frac{z_{b}}{x_{b}}\\ r=\sqrt{x_{b}^{2}+y_{b}^{2}+z_{b}^{2}} \end{array}$$

then the coordinate of target can be written as

(9) $$\left\{\begin{array}{c} x_{b}=r\cos\alpha \cos\beta \\ y_{b}=r\sin\alpha \\ z_{b}=-r\cos\alpha \sin\beta \end{array}\right.$$

Substitute (6) in (9)

(10) $$\left\{\begin{array}{c} x_{b}=\left(\tilde{r}-\Delta r\right)\cos\left(\tilde{\alpha }-\Delta \alpha \right)\cos\left(\tilde{\beta }-\Delta \beta \right)\\ y_{b}=\left(\tilde{r}-\Delta r\right)\sin\left(\tilde{\alpha }-\Delta \alpha \right)\\ z_{b}=-\left(\tilde{r}-\Delta r\right)\cos\left(\tilde{\alpha }-\Delta \alpha \right)\sin\left(\tilde{\beta }-\Delta \beta \right) \end{array}\right.$$

Let $X_{b}={\left[\begin{array}{ccc} x_{b} & y_{b} & z_{b} \end{array}\right]}^{T}$, $\xi ={\left[\begin{array}{ccc} \Delta r & \Delta \alpha & \Delta \beta \end{array}\right]}^{T}$. Since the measurement errors are small values as assumed, the Equation (10) can be expressed as the first-order linearity of the errors

(11) $$X_{b}={\tilde{X}}_{b}+J\xi$$

where ${\tilde{X}}_{b}$ is the reported coordinate of target, J is the Jacobian matrix of $X_{b}$ with respect to ξ given by

(12) $$J=\left[\begin{array}{ccc} \frac{\partial x_{b}}{\partial \Delta r} & \frac{\partial x_{b}}{\partial \Delta \alpha } & \frac{\partial x_{b}}{\partial \Delta \beta }\\ \frac{\partial y_{b}}{\partial \Delta r} & \frac{\partial y_{b}}{\partial \Delta \alpha } & \frac{\partial y_{b}}{\partial \Delta \beta }\\ \frac{\partial z_{b}}{\partial \Delta r} & \frac{\partial z_{b}}{\partial \Delta \alpha } & \frac{\partial z_{b}}{\partial \Delta \beta } \end{array}\right]$$

Now, here are two sensors A and B. ($\lambda_{a}$,$\varphi_{a}$,$h_{a}$) and ($\lambda_{b}$,$\varphi_{b}$,$h_{b}$) are their longitude, latitude and height coordinates. Let $X_{ea}={\left[\begin{array}{ccc} x_{ea} & y_{ea} & z_{ea} \end{array}\right]}^{T}$ and $X_{eb}={\left[\begin{array}{ccc} x_{eb} & y_{eb} & z_{eb} \end{array}\right]}^{T}$ be the ECEF coordinates of two sensors respectively and $X_{et}={\left[\begin{array}{ccc} x_{et} & y_{et} & z_{et} \end{array}\right]}^{T}$ represents the ECEF coordinate of target. $X_{ba}={\left[\begin{array}{ccc} x_{ba} & y_{ba} & z_{ba} \end{array}\right]}^{T}$ and $X_{bb}={\left[\begin{array}{ccc} x_{bb} & y_{bb} & z_{bb} \end{array}\right]}^{T}$ are the body coordinates of target from two sensors respectively. Thus, the following relationships are given.

(13) $$\left[\begin{array}{c} x_{et}\\ y_{et}\\ z_{et} \end{array}\right]=\left[\begin{array}{c} x_{ea}\\ y_{ea}\\ z_{ea} \end{array}\right]+\left[\begin{array}{ccc} -\sin\lambda_{a} & -\sin\varphi_{a}\cos\lambda_{a} & \cos\varphi_{a}cos\lambda_{a}\\ \cos\lambda_{a} & -\sin\varphi_{a}\sin\lambda_{a} & \cos\varphi_{a}\sin\lambda_{a}\\ 0 & \cos\varphi_{a} & \sin\varphi_{a} \end{array}\right]\left[\begin{array}{c} x_{ba}\\ y_{ba}\\ z_{ba} \end{array}\right]$$

(14) $$\left[\begin{array}{c} x_{et}\\ y_{et}\\ z_{et} \end{array}\right]=\left[\begin{array}{c} x_{eb}\\ y_{eb}\\ z_{eb} \end{array}\right]+\left[\begin{array}{ccc} -\sin\lambda_{b} & -\sin\varphi_{b}\cos\lambda_{b} & \cos\varphi_{b}cos\lambda_{b}\\ \cos\lambda_{b} & -\sin\varphi_{b}\sin\lambda_{b} & \cos\varphi_{b}\sin\lambda_{b}\\ 0 & \cos\varphi_{b} & \sin\varphi_{b} \end{array}\right]\left[\begin{array}{c} x_{bb}\\ y_{bb}\\ z_{bb} \end{array}\right]$$

From the two equations above, we obtain the following equality

(15) $$X_{ea}+R_{a}X_{ba}=X_{eb}+R_{b}X_{bb}$$

where $R_{a}$ and $R_{b}$ denote the rotational matrices in (13) and (14). $X_{ba}$ and $X_{bb}$ can be expanded to the linear form like (11), that is

(16) $$\begin{array}{cc} \left(X_{ea}+R_{a}{\tilde{X}}_{ba}\right)+R_{a}J_{a}\xi_{a} & =\left(X_{eb}+R_{b}{\tilde{X}}_{bb}\right)+R_{b}J_{b}\xi_{b}\\ {\tilde{X}}_{ea}-{\tilde{X}}_{eb} & =-R_{a}J_{a}\xi_{a}+R_{b}J_{b}\xi_{b} \end{array}$$

where ${\tilde{X}}_{ea}$ and ${\tilde{X}}_{eb}$ represent the ECEF coordinates of target reported by sensors A and B. In order to estimate the measurement errors of sensors A and B, the state vector is developed as

(17) $$\chi \left(k\right)={\left[\begin{array}{cccccc} \Delta r_{am} & \Delta \alpha_{am} & \Delta \beta_{am} & \Delta r_{bm} & \Delta \alpha_{bm} & \Delta \beta_{bm} \end{array}\right]}^{T}$$

where $\Delta r_{am}$, $\Delta \alpha_{am}$, and $\Delta \beta_{am}$ are the measurment biases of sensor A, $\Delta r_{bm}$, $\Delta \alpha_{bm}$, $\Delta \beta_{bm}$ denote the measurment biases of sensor B. Since these terms are constant, the discrete equation of the state can be written as

(18) $$\begin{array}{c} \chi \left(k\right)=F\left(k\right)\chi \left(k-1\right)+W\left(k\right)\\ F\left(k\right)={\left[\begin{array}{ccc} 1 & 0 & 0\\ 0 & \ddots & 0\\ 0 & 0 & 1 \end{array}\right]}_{6\times 6} \end{array}$$

where $W\left(k\right)$ is zero mean white Gaussian process noise with covariance $Q\left(k\right)$. $F\left(k\right)$ is the state transition matrix. In (16), let $Z={\tilde{X}}_{ea}-{\tilde{X}}_{eb}$, $H_{1}=-R_{a}J_{a}$, $H_{2}=R_{b}J_{b}$, $H=\left[\begin{array}{cc} H_{1} & H_{2} \end{array}\right]$. Therefore, the measurement equation can be expressed as

(19) $$Z\left(k\right)=H\left(k\right)\chi \left(k\right)+V\left(k\right)$$

where $V\left(k\right)$ is zero mean white Gaussian process noise with covariance $R\left(k\right)$. Kalman filtering techniques [27] using (18) and (19) can be applied to estimate sensor errors online.

### 3.3. Improved ECEF-KF Algorithm

From the above derivation, it can be seen that the traditional ECEF-KF method only fits for sensors on stationary base without attitude errors. For the sensors on high-speed moving missiles, the position’s and attitude’s change of missiles will influence the actual measurements, and the oriented biases of each partner which may cause the misalignment of tracking or even the loss of target must not be ignored. Considering this situation, an improved method making minor modifications on the original algorithm, is proposed to realize the simultaneous estimation of attitude and measurement errors.

Here assuming two missiles A and B as well. Considering the attitude change, (13) and (14) are modified as

(20) $$X_{et}=X_{ea}+C_{d,a}^{e}C_{b,a}^{d}X_{ba}$$

(21) $$X_{et}=X_{eb}+C_{d,b}^{e}C_{b,b}^{d}X_{bb}$$

where $C_{d,a}^{e}$ and $C_{d,b}^{e}$ equal with $R_{a}$ and $R_{b}$ in (15) are rotation matrices from the LENU coordinate system to the ECEF coordinate system (2) of missiles A and B. $C_{b,a}^{d}$ and $C_{b,b}^{d}$ represent rotation matrices from the body coordinate system to the LENU coordinate system (4). Combining the two equations above, there is

(22) $$\Delta X_{e}=X_{eb}-X_{ea}=C_{d,a}^{e}C_{b,a}^{d}X_{ba}-C_{d,b}^{e}C_{b,b}^{d}X_{bb}$$

Since the rotation matrix is orthogonal [28], (for one orthogonal matrix M, there is $M^{-1}=M^{T}$), (22) is transformed to

(23) $$X_{ba}={\left(C_{b,a}^{d}\right)}^{T}{\left(C_{d,a}^{e}\right)}^{T}\left(C_{d,b}^{e}C_{b,b}^{d}X_{bb}+\Delta X_{e}\right)$$

When attitude errors are small values, the rotation matrix from body coordinate system to LENU system can be written as [29]

(24) $$\begin{array}{c} {\tilde{C}}_{b}^{d}=\left(I-\phi \right)C_{b}^{d}\\ \phi =\left(\delta \times \right)=\left[\begin{array}{ccc} 0 & -\Delta \psi & \Delta \theta \\ \Delta \psi & 0 & -\Delta \gamma \\ -\Delta \theta & \Delta \gamma & 0 \end{array}\right]\\ \delta ={\left[\begin{array}{ccc} \Delta \psi & \Delta \theta & \Delta \gamma \end{array}\right]}^{T} \end{array}$$

Thus

(25) $$\begin{array}{c} C_{b,a}^{d}={\left(I-\phi_{a}\right)}^{T}{\tilde{C}}_{b,a}^{d}\\ C_{b,b}^{d}={\left(I-\phi_{b}\right)}^{T}{\tilde{C}}_{b,b}^{d} \end{array}$$

Substitute (25) in (23) and ignore the product of $\phi_{a}$ and $\phi_{b}$

(26) $$X_{ba}\approx {\tilde{C}}_{d}^{b,a}\left(\left(I-\phi_{a}\right)C_{e}^{d,a}C_{d,b}^{e}-C_{e}^{d,a}C_{d,b}^{e}{\phi_{b}}^{T}\right){\tilde{C}}_{b,b}^{d}X_{bb}+{\tilde{C}}_{d}^{b,a}\left(I-\phi_{a}\right)C_{e}^{d,a}\Delta X_{e}$$

Both sides of (26) are multiplied by $C_{d,a}^{e}{\tilde{C}}_{b,a}^{d}$

(27) $$C_{d,a}^{e}{\tilde{C}}_{b,a}^{d}X_{ba}=(C_{d,b}^{e}-C_{d,a}^{e}\phi_{a}C_{e}^{d,a}C_{d,b}^{e}-C_{d,b}^{e}{\phi_{b}}^{T}){\tilde{C}}_{b,b}^{d}X_{bb}+C_{d,a}^{e}\left(I-\phi_{a}\right)C_{e}^{d,a}\Delta X_{e}$$

Expand $X_{ba}$ and $X_{bb}$ into the form of first-order linearity as (11). Since the item ϕ and ξ are both small values, their product is ignored. Therefore, (27) becomes

(28) $$\begin{array}{c} C_{d,a}^{e}{\tilde{C}}_{b,a}^{d}{\tilde{X}}_{ba}-C_{d,b}^{e}{\tilde{C}}_{b,b}^{d}{\tilde{X}}_{bb}+\Delta X_{e}\approx C_{d,b}^{e}{\tilde{C}}_{b,b}^{d}J_{b}\xi_{b}\\ -C_{d,a}^{e}{\tilde{C}}_{b,a}^{d}J_{a}\xi_{a}-\left(C_{d,a}^{e}\phi_{a}C_{e}^{d,a}+C_{d,b}^{e}{\phi_{b}}^{T}C_{e}^{d,b}\right)C_{d,b}^{e}{\tilde{C}}_{b,b}^{d}{\tilde{X}}_{bb} \end{array}$$

In the above formula, the left part of the equation is labeled as $Z^{*}$. Let $H_{1}^{*}=-C_{d,a}^{e}{\tilde{C}}_{b,a}^{d}J_{a}$, $H_{2}^{*}=-C_{d,b}^{e}{\tilde{C}}_{b,b}^{d}J_{b}$ and the last term in the right part of the equation is represented as P, we arrive at

(29) $$Z^{*}=H_{1}^{*}\xi_{a}+H_{2}^{*}\xi_{b}+P$$

The following work is to transform P to the linear form of attitude errors. The matrix P is written as the following

(30) $$P=-(A\phi_{a}A^{T}+B\phi_{b}^{T}B^{T})M$$

where

$$\begin{array}{c} A=C_{d,a}^{e}=\left[\begin{array}{ccc} a_{1} & \;a_{2} & \;a_{3} \end{array}\right]\\ B=C_{d,b}^{e}=\left[\begin{array}{ccc} b_{1} & \;b_{2} & \;b_{3} \end{array}\right]\\ M=B{\tilde{C}}_{b,b}^{d}{\tilde{X}}_{bb} \end{array}$$

$a_{i}$ and $b_{i}$ are the column vectors of A and B respectively.

**Remark** **1.** *Here giving an equality:*

(31) $$A\phi_{a}A^{T}M=A{\left[\begin{array}{ccc} -a_{2} & \;a_{1} & \;0\\ a_{3} & \;0 & -a_{1}\\ 0 & -a_{3} & \;a_{2} \end{array}\right]}^{T}diag\left(M,M,M\right)\left[\begin{array}{c} \Delta \psi_{a}\\ \Delta \theta_{a}\\ \Delta \gamma_{a} \end{array}\right]$$

**Proof** **of** **Remark** **1.** $$\begin{array}{cc} Left & =\left[\begin{array}{ccc} a_{1} & a_{2} & a_{3} \end{array}\right]\left[\begin{array}{ccc} 0 & -\Delta \psi_{a} & \Delta \theta_{a}\\ \Delta \psi_{a} & 0 & -\Delta \gamma_{a}\\ -\Delta \theta_{a} & \Delta \gamma_{a} & 0 \end{array}\right]\left[\begin{array}{c} a_{1}^{T}\\ a_{2}^{T}\\ a_{3}^{T} \end{array}\right]M\\ & =\left[\begin{array}{ccc} a_{1} & a_{2} & a_{3} \end{array}\right]\left[\begin{array}{c} -\Delta \psi_{a}a_{2}^{T}+\Delta \theta_{a}a_{3}^{T}\\ \Delta \psi_{a}a_{1}^{T}-\Delta \gamma_{a}a_{3}^{T}\\ -\Delta \theta_{a}a_{1}^{T}+\Delta \gamma_{a}a_{2}^{T} \end{array}\right]M\\ & =\left(\left(a_{2}a_{1}^{T}-a_{1}a_{2}^{T}\right)\Delta \psi_{a}+\left(a_{1}a_{3}^{T}-a_{3}a_{1}^{T}\right)\Delta \theta_{a}+\left(a_{3}a_{2}^{T}-a_{2}a_{3}^{T}\right)\Delta \gamma_{a}\right)M\\ Right & =\left[\begin{array}{ccc} a_{1} & a_{2} & a_{3} \end{array}\right]\left[\begin{array}{ccc} -a_{2}^{T} & a_{3}^{T} & 0\\ a_{1}^{T} & 0 & -a_{3}^{T}\\ 0 & -a_{1}^{T} & a_{2}^{T} \end{array}\right]\left[\begin{array}{c} \Delta \psi_{a}M\\ \Delta \theta_{a}M\\ \Delta \gamma_{a}M \end{array}\right]\\ & =\left[\begin{array}{ccc} -a_{1}a_{2}^{T}+a_{2}a_{1}^{T} & a_{1}a_{3}^{T}-a_{3}a_{1}^{T} & -a_{2}a_{3}^{T}+a_{3}a_{2}^{T} \end{array}\right]\times \left[\begin{array}{c} \Delta \psi_{a}M\\ \Delta \theta_{a}M\\ \Delta \gamma_{a}M \end{array}\right]\\ & =Left \end{array}$$

So, the Theorem 1 is true. ☐

Likewise

(32) $$\begin{array}{c} B{\phi_{b}}^{T}B^{T}M=-B\phi_{b}B^{T}M\\ =-B{\left[\begin{array}{ccc} -b_{2} & b_{1} & \;0\\ b_{3} & \;0 & -b_{1}\\ 0 & -b_{3} & b_{2} \end{array}\right]}^{T}diag\left(M,M,M\right)\left[\begin{array}{c} \Delta \psi_{b}\\ \Delta \theta_{b}\\ \Delta \gamma_{b} \end{array}\right] \end{array}$$

Let

(33) $$\begin{array}{cc} H_{3}^{*} & =-A{\left[\begin{array}{ccc} -a_{2} & a_{1} & 0\\ a_{3} & 0 & -a_{1}\\ 0 & -a_{3} & a_{2} \end{array}\right]}^{T}diag\left(M,M,M\right)\\ H_{4}^{*} & =\;B{\left[\begin{array}{ccc} -b_{2} & b_{1} & 0\\ b_{3} & 0 & -b_{1}\\ 0 & -b_{3} & b_{2} \end{array}\right]}^{T}diag\left(M,M,M\right)\\ \delta_{a} & ={\left[\begin{array}{ccc} \Delta \psi_{a} & \Delta \theta_{a} & \Delta \gamma_{a} \end{array}\right]}^{T}\;\delta_{b}={\left[\begin{array}{ccc} \Delta \psi_{b} & \Delta \theta_{b} & \Delta \gamma_{b} \end{array}\right]}^{T} \end{array}$$

Substituting (31)∼(33) in (30), P is finally expanded as

(34) $$P\;=H_{3}^{*}\delta_{a}+H_{4}^{*}\delta_{b}$$

Let (34) in (29)

(35) $$\begin{array}{cc} Z^{*} & =H_{1}^{*}\xi_{a}+H_{2}^{*}\xi_{b}+H_{3}^{*}\delta_{a}+H_{4}^{*}\delta_{b}\\ & =H_{1}^{*}{\left[\begin{array}{ccc} \Delta r_{am}+\Delta r_{an} & \Delta \alpha_{am}+\Delta \alpha_{an} & \Delta \beta_{am}+\Delta \beta_{an} \end{array}\right]}^{T}\\ & +H_{2}^{*}{\left[\begin{array}{ccc} \Delta r_{bm}+\Delta r_{bn} & \Delta \alpha_{bm}+\Delta \alpha_{bn} & \Delta \beta_{bm}+\Delta \beta_{bn} \end{array}\right]}^{T}\\ & +H_{3}^{*}{\left[\begin{array}{ccc} \Delta \psi_{a} & \Delta \theta_{a} & \Delta \gamma_{a} \end{array}\right]}^{T}+H_{4}^{*}{\left[\begin{array}{ccc} \Delta \psi_{b} & \Delta \theta_{b} & \Delta \gamma_{b} \end{array}\right]}^{T} \end{array}$$

where $\left(\Delta r_{am},\Delta \alpha_{am},\Delta \beta_{am}\right),\left(\Delta r_{bm},\Delta \alpha_{bm},\Delta \beta_{bm}\right)$ are constant measurment biases of missile A and B respectively, $\left(\Delta r_{an},\Delta \alpha_{an},\Delta \beta_{an}\right),\left(\Delta r_{bn},\Delta \alpha_{bn},\Delta \beta_{bn}\right)$ are random noises added to these measurements.

Considering the terms of attitude errors, we redefine

(36) $$\chi^{*}\left(k\right)={\left[\Delta r_{am}\;\Delta \alpha_{am}\;\Delta \beta_{am}\;\Delta r_{bm}\;\Delta \alpha_{bm}\;\Delta \beta_{bm}\;\Delta \psi_{a}\;\Delta \theta_{a}\;\Delta \gamma_{a}\;\Delta \psi_{b}\;\Delta \theta_{b}\;\Delta \gamma_{b}\right]}^{T}$$

to be the 12-dimensional state vector. The corrected discrete equation of the state is

(37) $$\chi^{*}\left(k\right)=F^{*}\left(k\right)\chi^{*}\left(k-1\right)+W^{*}\left(k\right)$$

Since the attitude and measurement biases are all considered as constant values.

$$F^{*}\left(k\right)={\left[\begin{array}{ccc} 1 & 0 & 0\\ 0 & \ddots & 0\\ 0 & 0 & 1 \end{array}\right]}_{12\times 12}$$

Denoting $H^{*}=\left[\begin{array}{cccc} {H_{1}}^{*} & {H_{2}}^{*} & {H_{3}}^{*} & {H_{4}}^{*} \end{array}\right]$ In (35), the new measurement equation is

(38) $$Z^{*}\left(k\right)=H^{*}\left(k\right)\chi^{*}\left(k\right)+V^{*}\left(k\right)$$

where $V^{*}\left(k\right)$ is still zero mean white Gaussian process noise with covariance $R^{*}\left(k\right)$. Kalman filtering techniques using (37) and (38) can be used here as well, which is unnecessary to go into the details.

## 4. Target Tracking with Error Compensation

### 4.1. Compensation PLKF Algorithm

In Section 3, the coordinate of target in the body coordinate system has been given in (10):

(39) $$\left\{\begin{array}{c} x_{b}=\left(\tilde{r}-\Delta r\right)\cos\left(\tilde{\alpha }-\Delta \alpha \right)\cos\left(\tilde{\beta }-\Delta \beta \right)\\ y_{b}=\left(\tilde{r}-\Delta r\right)\sin\left(\tilde{\alpha }-\Delta \alpha \right)\\ z_{b}=-\left(\tilde{r}-\Delta r\right)\cos\left(\tilde{\alpha }-\Delta \alpha \right)\sin\left(\tilde{\beta }-\Delta \beta \right) \end{array}\right.$$

where $\Delta r\;=\Delta r_{m}\;+\Delta r_{n},\;\Delta \alpha =\Delta \alpha_{m}+\Delta \alpha_{n},\;\Delta \beta =\Delta \beta_{m}+\Delta \beta_{n}$.

In order to improve the accuracy of target tracking, the error compensation method is introduced.The estimation of registration errors including range $\Delta r_{m}^{*}$, elevation angle $\Delta \alpha_{m}^{*}$, azimuth angle $\Delta \beta_{m}^{*}$ and attitude errors $\Delta \psi^{*}$, $\Delta \theta^{*}$, $\Delta \gamma^{*}$ are obtained through ECEF-KF. These estimated values are compensated to the error terms of the equation above and the rotation matrix ${\tilde{C}}_{b}^{d}$ in (24), that is

(40) $$\Delta r_{m}\approx \Delta r_{m}^{*}\;\Delta \alpha_{m}\approx \Delta \alpha_{m}^{*}\;\Delta \beta_{m}\approx \Delta \beta_{m}^{*}$$

(41) $$C{{}_{b}^{d}}^{*}={\left(I-\phi^{*}\right)}^{T}{\tilde{C}}_{b}^{d}\;\phi^{*}=\left[\begin{array}{ccc} 0 & -\Delta \psi^{*} & \Delta \theta^{*}\\ \Delta \psi^{*} & 0 & -\Delta \gamma^{*}\\ -\Delta \theta^{*} & \Delta \gamma^{*} & 0 \end{array}\right]$$

Let $r^{\prime }=\tilde{r}-\Delta r_{m}^{*}$, $\alpha^{\prime }=\tilde{\alpha }-\Delta \alpha_{m}^{*}$, $\beta^{\prime }=\tilde{\beta }-\Delta \beta_{m}^{*}$, (39) becomes

(42) $$\left\{\begin{array}{c} x_{b}=\left(r^{\prime }-\Delta r_{n}\right)\cos\left(\alpha^{\prime }-\Delta \alpha_{n}\right)\cos\left(\beta^{\prime }-\Delta \beta_{n}\right)\\ y_{b}=\left(r^{\prime }-\Delta r_{n}\right)\sin\left(\alpha^{\prime }-\Delta \alpha_{n}\right)\\ z_{b}=-\left(r^{\prime }-\Delta r_{n}\right)\cos\left(\alpha^{\prime }-\Delta \alpha_{n}\right)\sin\left(\beta^{\prime }-\Delta \beta_{n}\right) \end{array}\right.$$

Considering that $\Delta r_{n},\Delta \alpha_{n},\Delta \beta_{n}$ are small values, there are $\cos\Delta \alpha_{n}\approx 1$, $\cos\Delta \beta_{n}\approx 1$, $\sin\Delta \alpha_{n}\approx \Delta \alpha_{n}$, $\sin\Delta \beta_{n}\approx \Delta \beta_{n}$. While, ignore the high-level small quantities, which means $\Delta \alpha_{n}\Delta \beta_{n}\approx 0$, $\Delta \alpha_{n}\Delta r_{n}\approx 0$, $\Delta r_{n}\Delta \beta_{n}\approx 0$. Then, the equation above turns to the following pseudo measurement:

(43) $$\left\{\begin{array}{c} l_{x}≜r^{\prime }\cos\alpha^{\prime }\cos\beta^{\prime }=x_{b}-n_{x}\\ l_{y}≜r^{\prime }\sin\alpha^{\prime }=y_{b}-n_{y}\\ l_{z}≜-r^{\prime }\cos\alpha^{\prime }\sin\beta^{\prime }=z_{b}-n_{z} \end{array}\right.$$

where $n_{x},n_{y},n_{z}$ are pseudo measurement noises, here are

$$\left\{\begin{array}{c} n_{x}=\Delta \beta_{n}r^{\prime }\cos\alpha^{\prime }\sin\beta^{\prime }+\Delta \alpha_{n}r^{\prime }\sin\alpha^{\prime }\cos\beta^{\prime }-\Delta r_{n}\cos\alpha^{\prime }\cos\beta^{\prime }\\ n_{y}=-\Delta \alpha_{n}r^{\prime }\cos\alpha^{\prime }-\Delta r_{n}\sin\alpha^{\prime }\\ n_{z}=\Delta \beta_{n}r^{\prime }\cos\alpha^{\prime }\cos\beta^{\prime }-\Delta \alpha_{n}r^{\prime }\sin\alpha^{\prime }\sin\beta^{\prime }+\Delta r_{n}\cos\alpha^{\prime }\sin\beta^{\prime } \end{array}\right.$$

The covariance of these measurement noises is

(44) $$R_{n}=\left[\begin{array}{ccc} Var\left(n_{x}\right) & Cov\left(n_{x},n_{y}\right) & Cov\left(n_{x},n_{z}\right)\\ Cov\left(n_{y},n_{x}\right) & Var\left(n_{y}\right) & Cov\left(n_{y},n_{z}\right)\\ Cov\left(n_{z},n_{x}\right) & Cov\left(n_{y},n_{z}\right) & Var\left(n_{z}\right) \end{array}\right]$$

where

$$\begin{array}{c} \sigma_{r}^{2}=Var\left(\Delta r_{n}\right),\sigma_{\alpha }^{2}=Var\left(\Delta \alpha_{n}\right),\sigma_{\beta }^{2}=Var\left(\Delta \beta_{n}\right)\\ \left\{\begin{array}{c} Var\left(n_{x}\right)={\left(\sigma_{\beta }r^{\prime }\cos\alpha^{\prime }\sin\beta^{\prime }\right)}^{2}+{\left(\sigma_{\alpha }r^{\prime }sin\alpha^{\prime }cos\beta^{\prime }\right)}^{2}+{\left(\sigma_{r}\cos\alpha^{\prime }cos\beta^{\prime }\right)}^{2}\\ Var\left(n_{y}\right)={\left(\sigma_{\alpha }r^{\prime }\cos\alpha^{\prime }\right)}^{2}+{\left(\sigma_{r}\sin\alpha^{\prime }\right)}^{2}\\ Var\left(n_{z}\right)={\left(\sigma_{\beta }r^{\prime }\cos\alpha^{\prime }cos\beta^{\prime }\right)}^{2}+{\left(\sigma_{\alpha }r^{\prime }sin\alpha^{\prime }sin\beta^{\prime }\right)}^{2}+{\left(\sigma_{r}\cos\alpha^{\prime }sin\beta^{\prime }\right)}^{2}\\ Cov\left(n_{x},n_{y}\right)=Cov\left(n_{y},n_{x}\right)=\cos\alpha^{\prime }\sin\alpha^{\prime }\cos\beta^{\prime }\times \left({\sigma_{r}}^{2}-{\left(r^{\prime }\sigma_{\alpha }\right)}^{2}\right)\\ Cov\left(n_{x},n_{z}\right)=Cov\left(n_{z},n_{x}\right)=\cos\beta^{\prime }\sin\beta^{\prime }\times \left({\left(r^{\prime }\cos\alpha^{\prime }\sigma_{\beta }\right)}^{2}-{\left(r^{\prime }sin\alpha^{\prime }\sigma_{\alpha }\right)}^{2}-{\left(\cos\alpha^{\prime }\sigma_{r}\right)}^{2}\right)\\ Cov\left(n_{y},n_{z}\right)=Cov\left(n_{z},n_{y}\right)=\cos\alpha^{\prime }\sin\alpha^{\prime }\sin\beta^{\prime }\times \left({\left(r^{\prime }\sigma_{\alpha }\right)}^{2}-{\sigma_{r}}^{2}\right) \end{array}\right. \end{array}$$

Representing $U={\left[\begin{array}{cccccc} x_{b} & y_{b} & z_{b} & {\dot{x}}_{b} & {\dot{y}}_{b} & {\dot{z}}_{b} \end{array}\right]}^{T}$ as the state vector, the continuous equation is

(45) $$\dot{U}=\mathrm{SU}+G\omega$$

where $\omega ={\left[\begin{array}{ccc} \omega_{x} & \omega_{y} & \omega_{z} \end{array}\right]}^{T}$ is zero-mean white noise with covariance Σ, and S is the transition matrix. The CV (Constant Velocity) model [30] is chosen as the target motion model, that is

$$S=\left[\begin{array}{cc} 0_{3} & I_{3}\\ 0_{3} & 0_{3} \end{array}\right]G=\left[\begin{array}{c} 0_{3}\\ I_{3} \end{array}\right]$$

The discrete-time state function is

(46) $$U\left(k\right)=T\left(k\right)U\left(k-1\right)+\Lambda \left(k\right)$$

where Λ(k) is discrete-time process noise, and the state transition matrix $T\left(k\right)=T\left(t_{k},t_{k+1}\right)$ at time $t_{k}$ over time interval $t_{\Delta }=t_{k+1}-t_{k}$ is given by

$$T\left(k\right)=e^{St_{\Delta }}\approx I+t_{\Delta }S$$

The covariance of the discrete-time process noise is

$$Q_{k}=E\left[\Lambda_{k}{\Lambda_{k}}^{T}\right]=\int_{t_{k}}^{t_{k+1}}T\left(t_{k},\tau \right)G\Sigma G^{T}T{\left(t_{k},\tau \right)}^{T}d\tau$$

Let $L={\left[\begin{array}{ccc} l_{x} & l_{y} & l_{z} \end{array}\right]}^{T}$ denotes the measurement vector. Therefore, the measurement equation is transformed to the linear function of the target state.

(47) $$\begin{array}{cc} L\left(k\right) & =Y\left(k\right)U\left(k\right)+\mu \left(k\right)\\ Y\left(k\right) & =\left[\begin{array}{cc} I_{3} & 0_{3} \end{array}\right] \end{array}$$

where $\mu \left(k\right)$ is zero-mean Gaussian noise.

### 4.2. Conpensation UKF Algorithm

If we directly take the compensation inputs as the measurements of filter $L^{*}={\left[r^{{}^{\prime }}\;\alpha^{{}^{\prime }}\;\beta^{{}^{\prime }}\right]}^{T}$, then the measurement equation will be changed as the non-linear form (8)

(48) $$L^{*}\left(k\right)=h\left(U\left(k\right)\right)+\mu^{*}\left(k\right)$$

where $\mu^{*}\left(k\right)$ is measurement noise with Ω(k) as variance. Combined with (46) and (48), the UKF algorithm for nonlinear system state estimation is exactly required. The formulas of UKF are directly given as follows.

Initializing:

(49) $$\begin{array}{c} \hat{U}\left(0\right)=E\left[U\left(0\right)\right]\\ P\left(0\right)=E\left[\left(U\left(0\right)-\hat{U}\left(0\right)\right){\left(U\left(0\right)-\hat{U}\left(0\right)\right)}^{T}\right] \end{array}$$

Computing sigma point set:

(50) $$\ell \left(k\right)=\left[\begin{array}{ccc} \hat{U}\left(k\right) & \hat{U}\left(k\right)+\sqrt{\left(n+\lambda \right)P\left(k\right)} & \hat{U}\left(k\right)-\sqrt{\left(n+\lambda \right)P\left(k\right)} \end{array}\right]$$

Prediction updating:

(51) $$\begin{array}{c} \ell \left(k+1|k\right)=T\left(k\right)\ell \left(k\right)\\ \hat{U}\left(k+1|k\right)=\sum_{i=0}^{2n}W_{i}^{m}\ell_{i}\left(k+1|k\right)\\ P\left(k+1|k\right)=\sum_{i=0}^{2n}W_{i}^{c}\left[\ell_{i}\left(k+1|k\right)-\hat{U}\left(k+1|k\right)\right]\times {\left[\ell_{i}\left(k+1|k\right)-\hat{U}\left(k+1|k\right)\right]}^{T}+Q\left(k+1\right)\\ {\hat{L}}_{i}^{*}\left(k+1|k\right)=h\left(\ell_{i}\left(k+1|k\right)\right)\\ {\hat{L}}^{*}\left(k+1|k\right)=\sum_{i=0}^{2n}W_{i}^{m}{\hat{L}}_{i}^{*}\left(k+1|k\right) \end{array}$$

Measurement updating:

(52) $$\begin{array}{c} P_{LL}\left(k+1\right)=\sum_{i=0}^{2n}W_{i}^{c}\left[{\hat{L}}_{i}^{*}\left(k+1|k\right)-{\hat{L}}^{*}\left(k+1|k\right)\right]{\left[{{\hat{L}}_{i}}^{*}\left(k+1|k\right)-{\hat{L}}^{*}\left(k+1|k\right)\right]}^{T}+\Omega \left(k+1\right)\\ P_{UL}\left(k+1\right)=\sum_{i=0}^{2n}W_{i}^{c}\left[\ell \left(k+1|k\right)-\hat{U}\left(k+1|k\right)\right]{\left[{\hat{L}}_{i}^{*}\left(k+1|k\right)-{\hat{L}}^{*}\left(k+1|k\right)\right]}^{T}\\ K\left(k+1\right)=P_{UL}\left(k+1\right){\left(P_{LL}\left(k+1\right)\right)}^{-1}\\ \hat{U}\left(k+1\right)=\hat{U}\left(k+1|k\right)+K\left(k+1\right)\left(L^{*}{\left(k+1|k\right)}_{k}-{\hat{L}}^{*}\left(k+1|k\right)\right)\\ P\left(k+1\right)=P\left(k+1|k\right)-K\left(k+1\right){\left(P_{LL}\left(k+1\right)\right)}^{-1}{\left(K\left(k+1\right)\right)}^{T} \end{array}$$

### 4.3. Compensation Condition and Strategy

**Remark** **2.** *Considering the situation where two missiles and target are on the same latitude or longitude plane during their fight, the 3-D tracking problem will become 2-D.*

**Figure 3:**
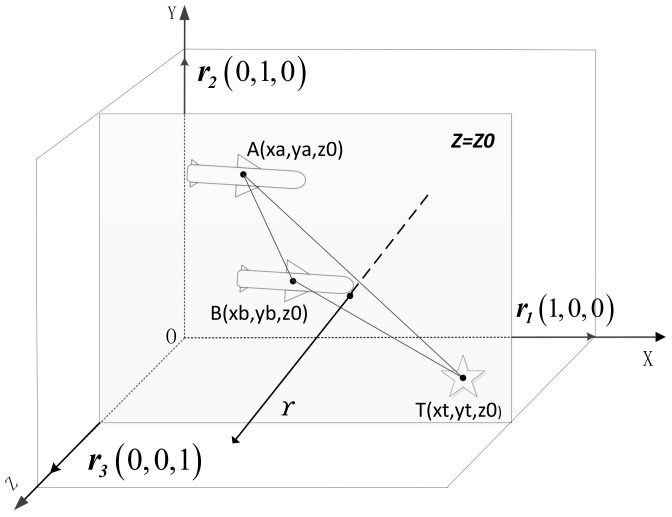
Judge on spatial relationship.

As shown in the Figure 3, OXYZ coordinate system is the longitude, latitude and height coordinate system. The axes OX, OY, OZ denote longitude, height and latitude respectively. In this moment, the two missiles and the target are on the same latitude plane $Z=Z_{0}$ and the track plane ABT is parallel to the plane OXY, which means the measurement of space registration will lost the measurements on OZ axis and the tracking problem in 3-D space turns to be on the plane OXY. If two missiles keep flying on this plane for some time, the estimation of errors may be divergent, which will worsen the compensation effect.

A judging condition is presented here: Assume Γ is the normal vector of the tracking plane ABT, $r_{i}$ is the unit vector parallel to the axes, $\vartheta_{i}$ is the vectorial angle between Γ and $r_{i}$.

(53) $$\left|\cos\vartheta_{i}-1\right|=\left|\frac{r_{i}\cdot \Gamma }{∥r_{i}∥∥\Gamma ∥}-1\right|\leq \varepsilon$$

where ε is a sufficiently small value, $∥\cdot ∥$ is the Euclidean metric of vector [31]. If the inequality satisfies, the situation in Remark 2 will happen.

To avoid the decreasing precision of compensation tracking, the estimation of registration errors will be introduced to a LFOF when (53) is true, that is

(54) $$y=\frac{1}{\kappa s+1}u\Rightarrow \dot{y}=-\frac{1}{\kappa }y+\frac{1}{\kappa }u$$

where *u* is the error items, *y* is the output of the filter, κ is the propotionality coefficient. The fourth-order Runge-Kutta algorithm [32,33] can be used to solve the above differential equation.

**Figure 4:**
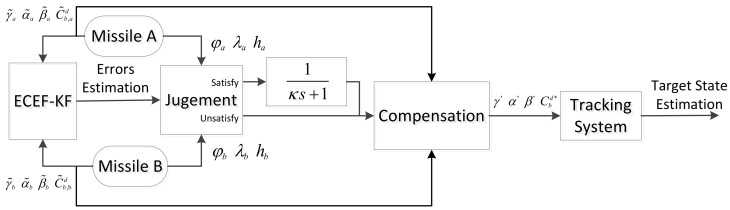
Arcitecture of registration and tracking algorithm.

Figure 4 shows the flow of whole algorithms. Before target tracking, the ECEF-KF is introduced to obtain registration errors of measurements of missiles. The errors acquired are furtherly introduced to the tracking system through the conditional compensation. When the tracking plane is parallel to one of three coordinate planes, the LFOF is taken to inhibit the divergence of error estimations.

The compensation PLKF method with LFOF is called the CCPLKF (Conditional Compensation Pseudo Linear Kalman Filter), otherwise, called UCPLKF (Unconditional Compensation Pseudo Linear Kalman Filter). Accordingly, the compensation UKF method is divided to CCUKF and UCUKF.

## 5. Simulation

In this section, the performance of the proposed algorithm in spatial registration compared with traditional ECEF-KF is demonstrated. And the target tracking scheme is proved effective through the comparison of linear filters and nonlinear filters.

In the simulation, the ground target on curvilinear motion with variable velocity is considered. The velocity of target is (N 10 sin$\frac{2\pi t}{T}$, E 5m/s), wherein *t* is the current time and *T* is the total simulation time.

The initial position of target is (longitude $108.5^{\circ }$, latitude $34.01^{\circ }$, height 0 m). The same terms of 1th and 2th missiles are (longitude $108^{\circ }$, latitude $34^{\circ }$, height 300 m) and (longitude $108^{\circ }$, latitude $34.02^{\circ }$, height 200 m) respectively. There are two motion situations of missiles being considered:

Case 1: Both two missiles make uniform linear flight to the east, the velocity are (N 0 m/s, E 120 m/s) and (N 0 m/s, E 100 m/s) respectively.

**Figure 5:**
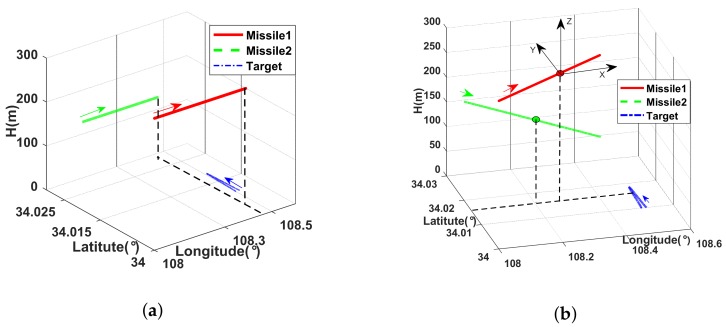
(**a**) Trajectories of missiles and target under case 1; (**b**)Trajectories of missiles and target under case 2.

Case 2: The missiles make uniform linear flight as well, the velocity are (N 5 m/s, E 120 m/s) and (N −7 m/s, E 100 m/s) respectively. Figure 5 shows the absolute trajectories of missiles and target in the longitude, latitude and height coordinate system under the cases above.

The 1th sensor offset error is (range 200 m, elevation angle $0.5^{\circ }$, azimuth angle $0.2^{\circ }$) and the 2th sensor offset error is (range 300 m, elevation angle $0.3^{\circ }$, azimuth angle $0.2^{\circ }$). Both two sensors’ standard deviation of measurement noises is (5 m, $0.01^{\circ }$, $0.1^{\circ }$) All the measurement errors and noises are added to ideal measurements to create reported values. Both two platforms’ initial attitudes are (yaw $1^{\circ }$, pitch $0^{\circ }$, roll $3^{\circ }$) and offset errors of these angles are $0.01^{\circ }$, which are added to initial attitudes to create deviated values. The sampling interval is 0.1 s. All the simulation results are obtained over 100 Monte Carlo trials. The performance of improved ECEF-KF algorithm proposed in the paper is compared with the method based on ECEF-LS in [24] and the standard Kalman filter, which is called traditional ECEF-KF.

**Figure 6:**
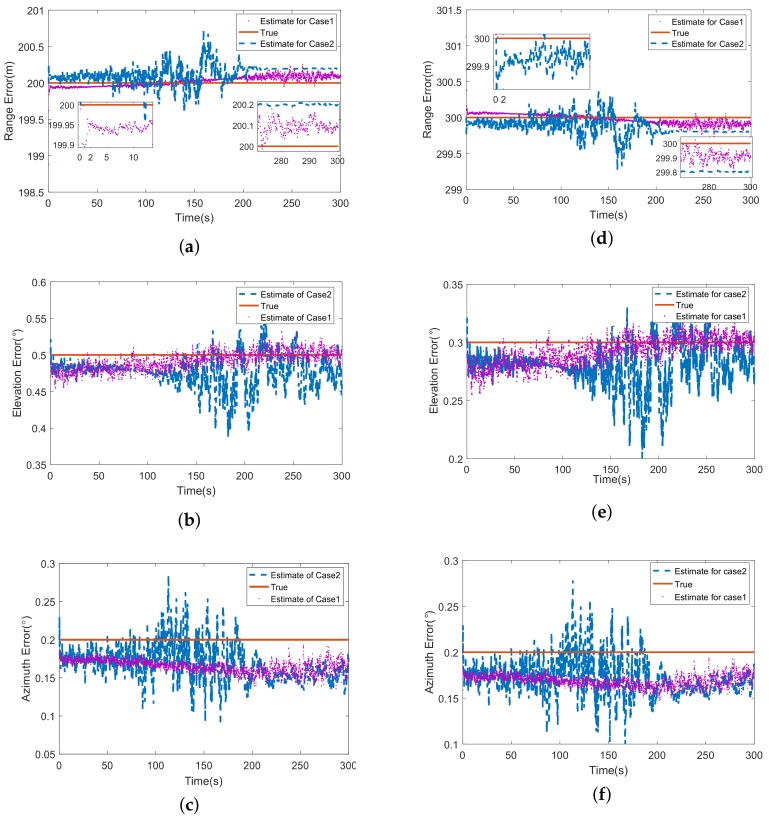
(**a**) The estimation of range error of missile 1; (**b**) The estimation of elevation error of missile 1; (**c**) The estimation of azimuth error of missile 1; (**d**) The estimation of range error of missile 2; (**e**) The estimation of elevation error of missile 2; (**f**) The estimation of azimuth error of missile 2.

The estimations of sensor errors are proposed in Figure 6, wherein the left column is about missile 1 and the right column presents the outcome of missile 2. The results of two cases are compared in one figure. It is obvious that the curves of case 2 jitter severely during 100∼200 s, which does not appear in the case 1. As mentioned in Section 4.2, the jitter is caused by the approach of latitudes of two missiles and target (see in Figure 5b). At 150 s, the two missiles move to nearly the same latitude (about $34.01^{\circ }$), the latitude of target is about $34.0186^{\circ }$, they are almost on the same latitude plane. That means the measurements of ECEF-KF become two dimensional, which causes the divergence during this time and converge gradually with the continual motion of missiles.

**Figure 7:**
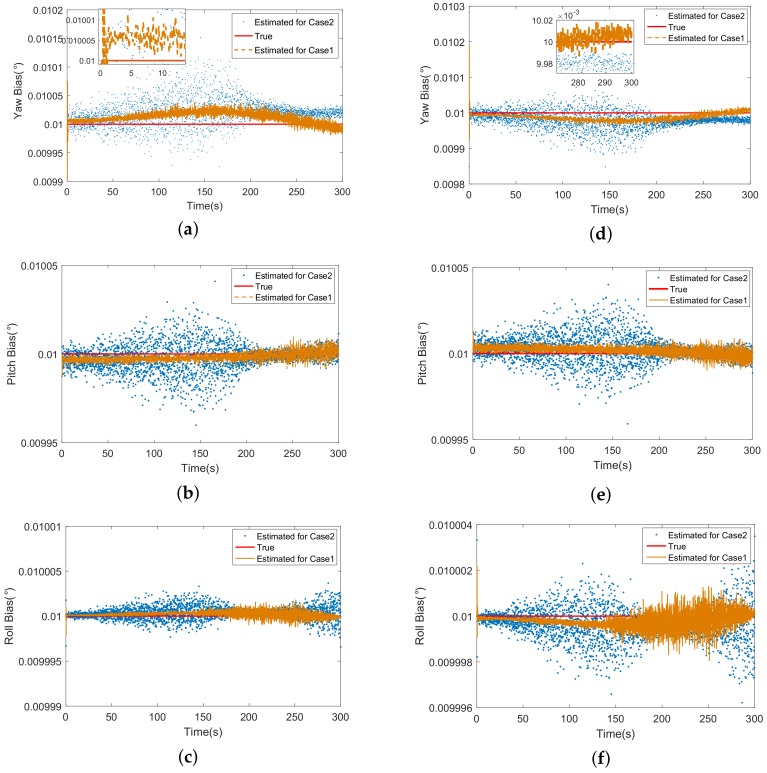
(**a**) The estimation of yaw bias of missile 1; (**b**) The estimation of pitch bias of missile 1; (**c**) The estimation of roll bias of missile 1; (**d**) The estimation of yaw bias of missile 2; (**e**) The estimation of elevation error of pitch bias of missile 2; (**f**) The estimation of roll bias of missile 2.

The Figure 7 shows the estimations of attitude biases. The divergency during 100∼200 s appears similarly under case 2. Besides, it is found in Figure 7c,f that the roll error increases after 200 s. This is because the longitudes of missiles come close to the targets’ at the end of track, which leads to the same phenomenon caused by the approach of latitude. Since the measurement equation of ECEF-KF is a first-order linear function of state, the estimations converge quickly from beginning (about 2 s in Figure 7a) and jitter near the truth.

**Figure 8:**
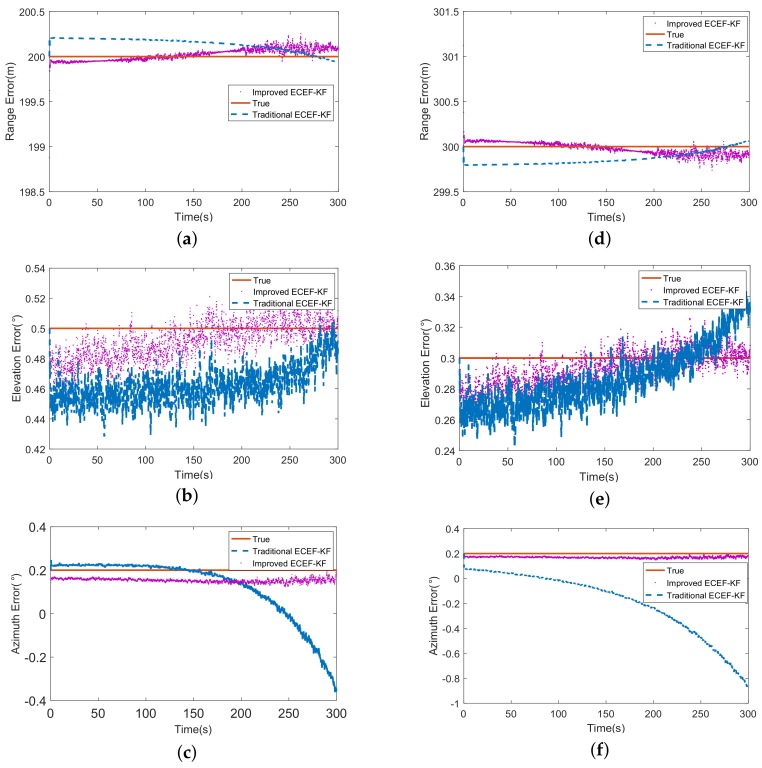
(**a**) The comparison of range error of missile 1; (**b**) The comparison of elevation error of missile 1; (**c**) The comparison of azimuth error of missile 1; (**d**) The comparison of range error of missile 2; (**e**) The comparison of elevation error of missile 2; (**f**) The comparison of azimuth error of missile 2.

To demonstrate the priority of proposed registration method for sensors on motivated platforms, Figure 8 illustrates the bias estimates of traditional ECEF-KF and improved one under case 1. The overlook of attitudes of orientated frames makes the former deviate from the truth as well as the disability to estimate the align errors. On the contrary, the latter which follows the truth closely gives better performance on the results. The reason causing the disappointment of traditional one is that the attitudes of missiles lead to the noncoincidence of LENU system and missile body system and influence the measurement obtained from sensors. For instance, the positive yaw value would increase the measurement of azimuth angle which is actually invariant in LENU system. However, the item $C_{b}^{d}$ of improved method transform the measurement from body system to LENU system, isolating the disturbance of frame angles.

The RMSE (Root Mean Square Error) of the estimation of two methods is listed in Table 1. Obviously, the improved ECEF-KF outperforms the original strategy in all terms, especially for the azimuth because of the relatively large value of yaw angle, which demonstrates the proposed algorithm has more advantage in spatial registration for mobile sensors.

**Figure 9:**
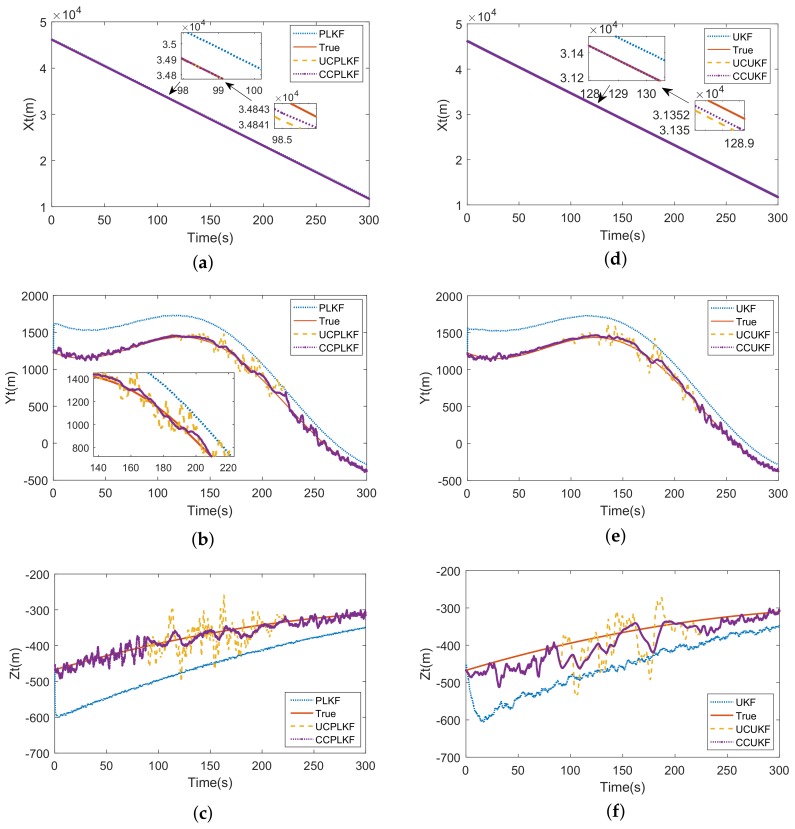
(**a**) Linear estimation of *x* coordinate of target; (**b**) Linear estimation of *y* coordinate of target; (**c**) Linear estimation of *z* coordinate of target; (**d**) Nonlinear estimation of *x* coordinate of target; (**e**) Nonlinear estimation of *y* coordinate of target; (**f**) Nonlinear estimation of *z* coordinate of target.

Take case 2 for example, the target tracking is conducted on missile 1. Figure 9 compares the estimations of different schemes, wherein the left column presents the results of linear filters and the nonlinear filters are illustrated in the right list. The trajectory of target is estimated in the LENU coordinate system of missile 1 (see in Figure 5b). The parameters are given ε=0.001,κ=10.

**Remark** **3.** *It is notable that ε is the limit of (53) and κ is the scale factor of integrator in (54). The former wherein will influence the time of error compensation (the lager ε is, the longer the LFOF will be taken) and the latter wherein will enhance the stability of error estimations (the lager κ is, the smoother the estimation through LFOF will be).*

Seen from the left column of Figure 9, the accuracy of estimation dramatically improved after compensation (the estimation of UCPLKF is much closer to the truth value than PLKF). This is because the constant biases are subtracted from the input of UCPLKF. But during 100∼200 s, the estimations of these biases deviate from the truth, resulting to the unsatisfactory performance of UCPLKF in this period. The CCPLKF which can effectively inhibit the jitter of curves through a condition-based LFOF is prior to UCPLKF. Since the LFOF only works when (53) satisfies, so it does not cause the time delay to the whole tracking process. In the right column, likewise, the CCUKF is the optimal method among the nonlinear tracking schemes.

**Figure 10:**
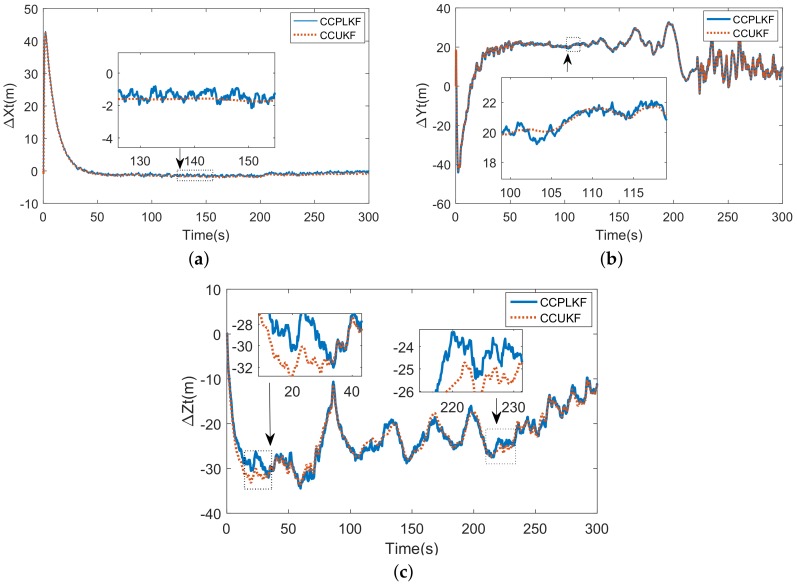
(**a**) Estimation Error of *x* coordinate of target; (**b**) Estimation Error of *y* coordinate of target. (**c**) Estimation Error of *z* coordinate of target.

The RMSE of the estimation of target state is listed in Table 2. Seen from the results, the accuracy of tracking system can be greatly improved through compensation and the conditional compensation methods (CCPLKF and CCUKF) have nearly the same and the smallest errors. The CCUKF although has slight edge over CCPLKF in the items RMSEx and RMSEy, but suffer from more error in RMSEz, which turns the advantage to disadvantage in joint distance. This result can be explained through the comparison of Figure 9c,f. As mentioned, the registration errors jitter severely during 100∼200 s, which are compensated directly to the measurement inputs of UKF yet indirectly introduced to PLKF. Thus, the latter has the better input-jitter tolerance, which can also be proved in Figure 10.

The estimations of CCPLKF and CCUKF are compared in Figure 10. Although the local magnifications in Figure 10a,b show the smoother curves of CCUKF than those of CCPLKF, but the Figure 10c exposes its flaw when the inputs jitter more severely. Moreover, the another fatal weakness of CCUKF is its relatively large computation. Given from simulation, the operation time caused by CCPLKF is 2.32 s, whereas CCUKF takes 5.62 s. In summary, the CCPLKF method keeps the best performance in target tracking.

## 6. Conclusions

In this paper, a novel spatial registration algorithm is proposed to estimate biases of measurements and orientation angles simultaneously. The linear and nonlinear filtering algorithms are introduced to solve the three-dimensional maneuvering target tracking problem. The estimations of registration method are used to modify the inputs of tracking scheme. In addition, a low pass first order filter is selectively taken to inhibit the jitter of estimation without the time delay. Simulation shows the good performance and robustness of the registration approach and the advantage of linear tracking method with modified inputs. Research results of this paper can also be extended to solve the registration and tracking of a dynamic network consisted of large quantities of sensors and multi-maneuvering targets [34,35].

## Figures and Tables

**Table 1:** RMSE of registration errors.

Method	Platform	Range (m)	Elevation (${}^{\circ }$)	Azimuth (${}^{\circ }$)
Improved	Missile 1	0.1878	0.0126	0.0327
ECEF-KF	Missile 2	0.1888	0.0057	0.0255
Traditional	Missile 1	0.2667	0.0522	0.5557
ECEF-KF	Missile 2	0.2688	0.0475	1.0674

**Table 2:** RMSE of tracking errors.

Method	RMSEx (m)	RMSEy (m)	RMSEz (m)	RMSEr (m)
PLKF	191.6083	269.8833	88.9778	342.6377
UCPLKF	6.3140	20.5581	24.1859	32.4938
CCPLKF	6.2862	18.8728	23.5337	30.9746
UKF	191.3337	269.8901	89.1703	342.7357
UCUKF	6.2568	20.5293	24.3977	32.3645
CCUKF	6.2366	18.8422	23.7803	30.8145
